# A Multimodal Classification Architecture for the Severity Diagnosis of Glaucoma Based on Deep Learning

**DOI:** 10.3389/fnins.2022.939472

**Published:** 2022-06-29

**Authors:** Sanli Yi, Gang Zhang, Chaoxu Qian, YunQing Lu, Hua Zhong, Jianfeng He

**Affiliations:** ^1^School of Information Engineering and Automation, Kunming University of Science and Technology, Kunming, China; ^2^First Affiliated Hospital of Kunming Medical University, Kunming, China

**Keywords:** glaucoma, computer-aided diagnosis, multimodal fusion, classification, multi-layer perceptron

## Abstract

Glaucoma is an optic neuropathy that leads to characteristic visual field defects. However, there is no cure for glaucoma, so the diagnosis of its severity is essential for its prevention. In this paper, we propose a multimodal classification architecture based on deep learning for the severity diagnosis of glaucoma. In this architecture, a gray scale image of the visual field is first reconstructed with a higher resolution in the preprocessing stage, and more subtle feature information is provided for glaucoma diagnosis. We then use multimodal fusion technology to integrate fundus images and gray scale images of the visual field as the input of this architecture. Finally, the inherent limitation of convolutional neural networks (CNNs) is addressed by replacing the original classifier with the proposed classifier. Our architecture is trained and tested on the datasets provided by the First Affiliated Hospital of Kunming Medical University, and the results show that the proposed architecture achieves superior performance for glaucoma diagnosis.

## Introductions

Glaucoma is a major eye health problem that leads to irreversible visual impairment (Mirzania et al., 2020). Because glaucoma initially tends to affect marginal vision and may still be asymptomatic until the middle stage, most patients are not treated in time, and further damage can occur (Yang et al., 2020). Thus, the detection and especially the severity classification of glaucoma is beneficial for ophthalmologists to analyze the condition of patients and develop follow-up treatment plans.

Fundus images, optical coherence tomography (OCT), and visual field are used as public data in the clinic. OCT can accurately evaluate the thickness of the retinal nerve fiber layer (RNFL) by tomography technology (Bowd et al., 2022). Fundus images reflect the vascular status of the eyes by contrast agent injection, and Chan et al. (2014) demonstrated that mono fundus images can provide an equal diagnostic accuracy for glaucomatous optic neuropathy evaluation when compared to stereoscopic images. The gray scale image of the visual field manifests the defect of the patient's visual field by brightness transformation (Wroblewski et al., 2009). Compared with OCT, fundus images and visual fields are easier to obtain and can be directly used to diagnose glaucoma (Wroblewski et al., 2009; Chan et al., 2014). The diagnosis of pathological images is crucial but time-consuming and laborious; thus, reliable computer-assisted diagnosis (CAD) of glaucoma has continued to expand in the recent years (Zheng et al., 2019). The diagnostic approaches by the above technologies for glaucoma can be divided into two categories. One is the single-path method, of which the input is single type data. For example, Wroblewski et al. (2009) used support vector machines (SVMs) to provide a valid clinical diagnosis of glaucoma based solely on visual field data. Escamez et al. (2021) developed a classifier for predicting glaucoma eyes based on peripapillary retinal nerve fiber layer (RNFL) thicknesses measured with OCT. The other is a multimodal fusion image, which is a combination of two or more types of data. For instance, Bizios et al. (2011) and Chen et al. (2019) employed multimodal fusion approaches to diagnose glaucoma by integrating OCT and visual field data and OCT and fundus images.

Nevertheless, there are at least three problems to be resolved. First, the inferior resolution of the common gray scale of the visual field affects the feature extraction of convolutional neural networks (CNNs) in the task of glaucoma diagnosis. Second, the majority of studies focused on employing a single type of data to simply diagnose health and glaucoma, whereas the diagnosis of glaucomatous severity is more significant for ophthalmologists (Rajendrababu et al., 2021). Third, some studies using CNNs to capture features still had difficulty meeting the requirements of accuracy in practical diagnostic tasks. The main reason is that each convolution kernel of CNNs focuses only on the feature information of itself and its boundary while lacking the ability to model some long-range dependencies in glaucoma images (Yao et al., 2021).

To address these challenges, we propose a multimodal classification architecture based on deep learning for the severity classification of glaucoma. In this architecture, first, the gray scale image of the visual field is reconstructed with a higher resolution in the preprocessing stage, which is conducive to the feature extraction of the proposed architecture. Second, the fundus image and reconstructed visual field gray scale image are integrated to obtain multimodel information for the classification task and then transferred into CNN models for feature extraction. Third, we construct an efficient classifier to address the limitation of CNNs. This adopts the multilayer perceptron (MLP) of vision transformer (Dosovitskiy et al., 2020) (ViT) to further extract global sequence information and can be directly connected after CNNs to replace its original classifier. The main contributions of this paper are as follows:

- A multimodal classification architecture based on deep learning is constructed for the task of severity classification of glaucoma. The gray scale image of the visual field is reconstructed with a higher resolution in the preprocessing stage, in which a more subtle gray scale division unit is modeled to provide more detailed feature information in the glaucoma diagnosis task.
- The proposed architecture fuses the fundus image and visual field gray scale image as the input to provide more information for the feature extraction of the network. This architecture realizes a 4-classification of glaucoma to present its severity, which is more convenient for ophthalmologists.
- To offset the limitation of CNNs, we propose a plug-and-play classifier which adopts the multilayer perceptron (MLP) of ViT to extract the global dependencies of images. Meanwhile, the proposed classifier can easily replace the original classifier of CNNs and significantly improve the accuracy of the diagnostic task.

## Background and Related Works

In this section, the latest progress of deep learning and its application in the field of glaucoma diagnosis are reviewed.

### Development of Deep Learning

In the recent years, deep learning algorithms, especially CNNs, have made significant progress. The introduction of ImageNet (Krizhevsky et al., 2017) provided an initial explanation for the conception of deep learning. Subsequently, Simonyan and Zisserman (2014) and Iandola et al. (2017) proposed visual geometry group (VGG) and SqueezeNet, respectively; they increased the depth of the network while keeping the perception field unchanged and improving the performance of the networks. Meanwhile, He et al. (2016) and Huang et al. (2016) introduced functional modules such as residual and dense modules to enhance the performance of CNNs. Due to these improvements, CNNs are widely applied in the field of CAD. However, CNNs lack the ability to model the global dependencies of images because of their inherent limitations. Recently, transformer (Vaswani et al., 2017), which is capable of modeling long-range sequence features, attracted tremendous attention in the computer vision field. Dosovitskiy et al. (2020) introduced a transformer into the image task and successfully used embedded 2-dimensional (2D) image patches as an input sequence to achieve comparable representation with CNNs. Therefore, to obtain better performance in the task of glaucoma diagnosis, it will be of greater significance to combine transformer to offset the limitations of the CNN model.

### Deep Learning for Glaucoma Diagnosis

Many deep learning algorithms have been employed in the fields of glaucomatous classification (Gour and Khanna, 2020; Wang et al., 2020; Singh et al., 2021). Raja et al. (2020) used a CNN to segment the retinal layer based on OCT data and calculate the cup-to-disk ratio (CDR). This achieved 94.6% accuracy in the glaucoma prediction task. Li et al. (2019) employed visual field data collected from hospitals to identify glaucoma, and the accuracy reached 87.6%. Kim et al. (2018) and Guo et al. (2020) diagnosed and localized fundus images by VGG16 and UNet++ networks to classify glaucoma and achieved an accuracy of 91.2% and an area under the curve (AUC) of 90.1%, respectively. Bajwa et al. (2020) and Ibrahim et al. (2022) both proposed a two-stage framework: the former detected and located optic disks on fundus images and then classified them as healthy or glaucoma; the latter preprocessed glaucoma disease data by normalization and the mean absolute deviation method in the first stage and trained a deep learning model through the artificial algae optimization algorithm later. They achieved an AUC of 87.4% and an F1 score of 98.15%.

Different from the above works, Bizios et al. (2011) used a multimodal fusion approach to diagnose glaucoma by fusing OCT and standard automated visual field data and improved the AUC by 3.3% compared with single data. Chen et al. (2019) employed residual UNet to segment enhanced OCT and fundus images and then integrated the extracted features, achieving an accuracy rate of 96.88%. Kang et al. (2020) fused cup-to-disk and retinal nerve fiber layer features for the diagnosis of glaucoma. In the work of Liu et al. (2014), the limitation of the performance of a single modality was overcome by integrating patient personal data, major ocular image features, and important genome SNP features. This approach obtained the best AUC compared with a single modality.

## Materials and Methods

**Figure 1:**
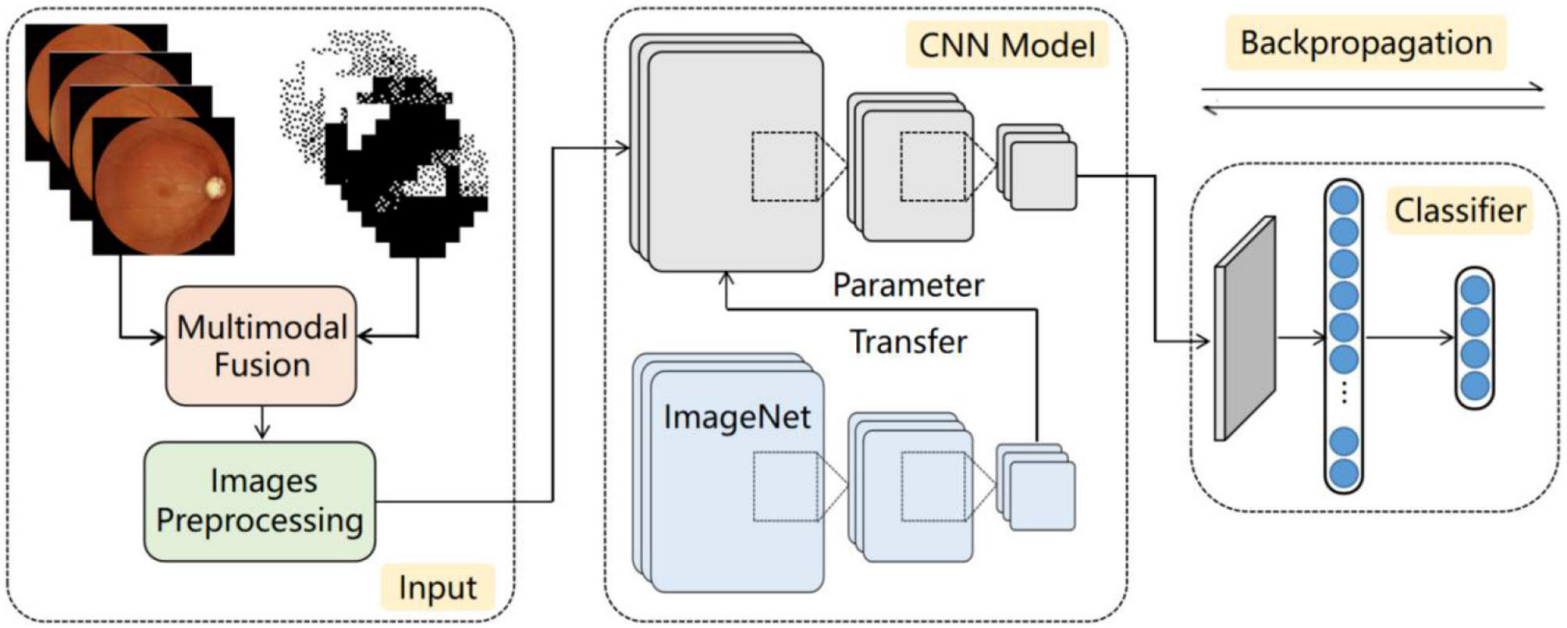
Diagram of proposed architecture.

The workflow of the proposed multimodal classification architecture is shown in Figure 1 and has three parts: input, CNN model, and classifier. First, the fundus image and reconstructed gray scale image of the visual field are fused into a multimodal fusion image, which are preprocessed and then sent into the CNN model. Second, as the feature extraction backbone of our architecture, the CNN model uses four ordinary CNNs to extract the feature information of the input image. These CNNs are pretrained by transfer learning technology to adapt to the task of small-scale datasets. Finally, the global dependencies of the feature maps are extracted by the proposed classifier to offset the limitations of the CNNs.

### Input

#### Datasets

**Figure 2:**
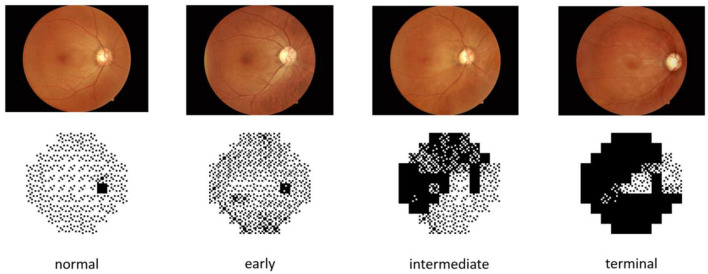
Samples of different severities.

The dataset of this paper is provided by the First Affiliated Hospital of Kunming Medical University. It contains 502 fundus images and 502 visual field reports from 274 individuals, and both eyes of each individual were used in the study. Fundus images and visual field reports were acquired by a Topcon fundus camera TRC-50DX and Intelligent Video Surveillance (ISV) automatic computerized perimetry, and each image was labeled by two professional physicians. The datasets were rated from class 0 to 3 based on the severity of glaucoma, representing normal (*n* = 87), early (*n* = 171), intermediate (*n* = 79), and terminal glaucoma (*n* = 165), respectively. Related information of the dataset is listed in Table 1. Meanwhile, to overcome the challenges of training on imbalanced data by CNNs, we augmented normal eyes from 87 to 174 and intermediate glaucoma from 79 to 158 through data augmentation technology and balanced the ratio of all categories of data to ~1:1:1:1. Finally, 1,336 images of the two types of data were applied to our deep learning architecture. The data sample is depicted in Figure 2.

**Table 1:** Distribution of dataset.

		**Normal (class 0)**	**Early (class 1)**	**Intermediate (class 2)**	**Terminal (class 3)**
Quantity	Original	87	171	79	165
	augmented	174	171	158	165

#### Preprocessing

The preprocessing consists of two parts: data augmentation and normalization, and improving the resolution of the visual field gray scale image by reconstructing gray scale units.

##### Augmentation and Normalization

As shown in Table 1, the distribution of each category in the dataset is severely imbalanced, which may skew the diagnosis of CNNs toward more data-intensive types. To address this problem, we use data augmentation technology such as rotation, flipping, brightness, and contrast adjustment to form a dataset with the sample number of each category being almost equal. Meanwhile, to make the data more suitable for the pretraining of CNNs based on ImageNet, of which the default input resolution is 224 × 224, the images are resized to 224 × 224 pixels by bilinear interpolation.

##### Reconstruction of Visual Field Gray Scale Images

**Figure 3:**
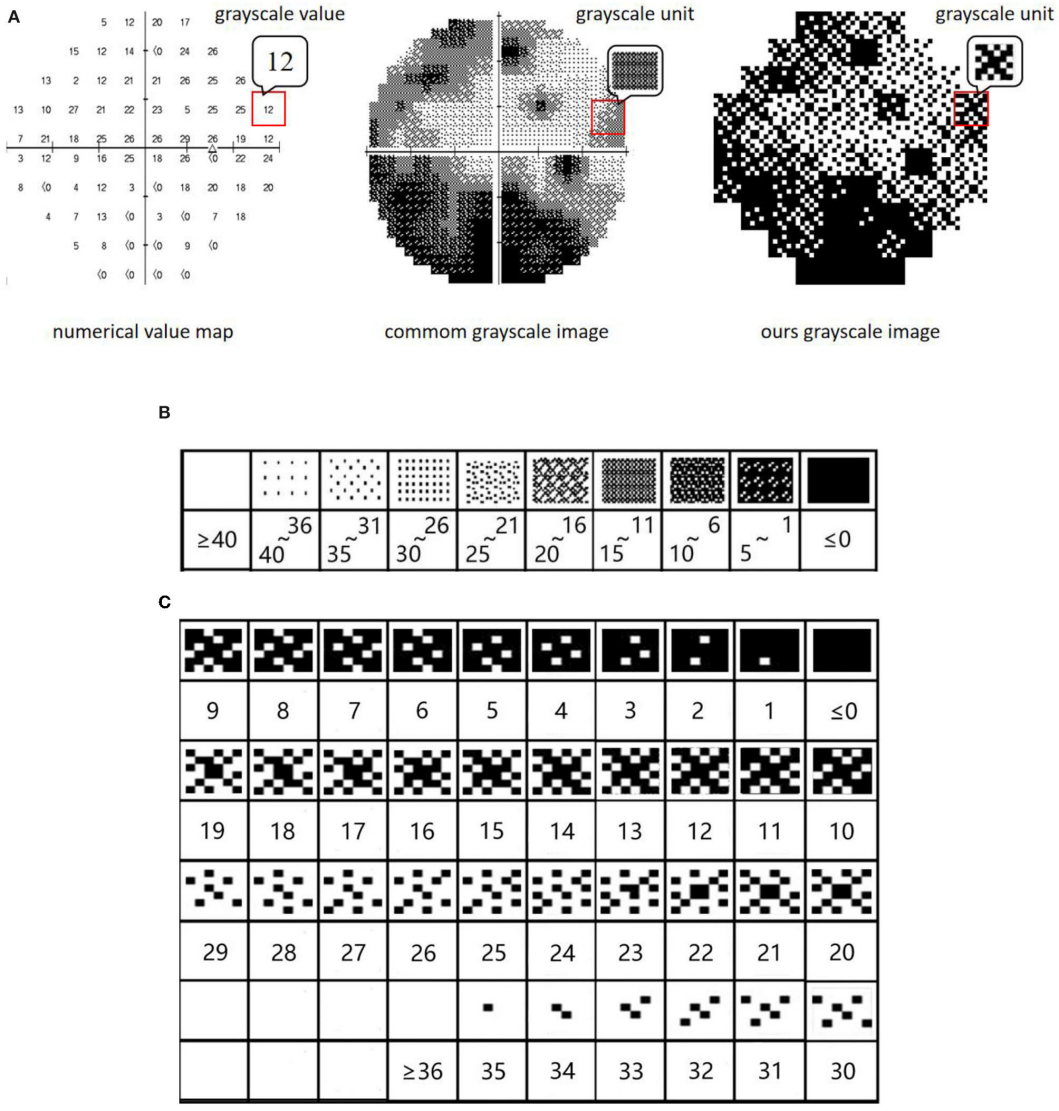
**(A)** Gray scale images of visual field. **(B)** Ordinary gray scale units. **(C)** New gray scale units.

As depicted in Figure 3A, the gray scale image of the visual field is constructed based on the numerical value map, and each gray scale value in the image is represented by a gray scale unit. In the ordinary gray scale image, due to its low resolution (each gray scale unit represents a value with a span of 5 dB) (Figure 3B), much information is lost in the training process of CNNs, thus affecting the ability of CNNs to extract subtle features. In this paper, to solve this problem, a more subtle gray scale unit and corresponding gray scale image are established in which the gray scale unit is divided into 1 dB to retain the subtle features of the gray scale image (Figure 3C).

##### Multimodal Fusion

In this paper, the proposed multimodal classification architecture fuses fundus images and visual field gray scale images through an image concatenation approach and then transfers it into the CNN model to capture sufficient feature information. This is different from other studies. For instance, Chen et al. (2019) input images into CNNs for extracting features and then fused the extracted features to diagnose glaucoma. Such a fusion method changes the extracted features during the fusion, so the fused feature information is not reliable. Our proposed architecture fuses multimodal images before training, avoiding the mutual interference of features while improving the performance of glaucoma diagnosis.

### CNN Model

**Figure 4:**
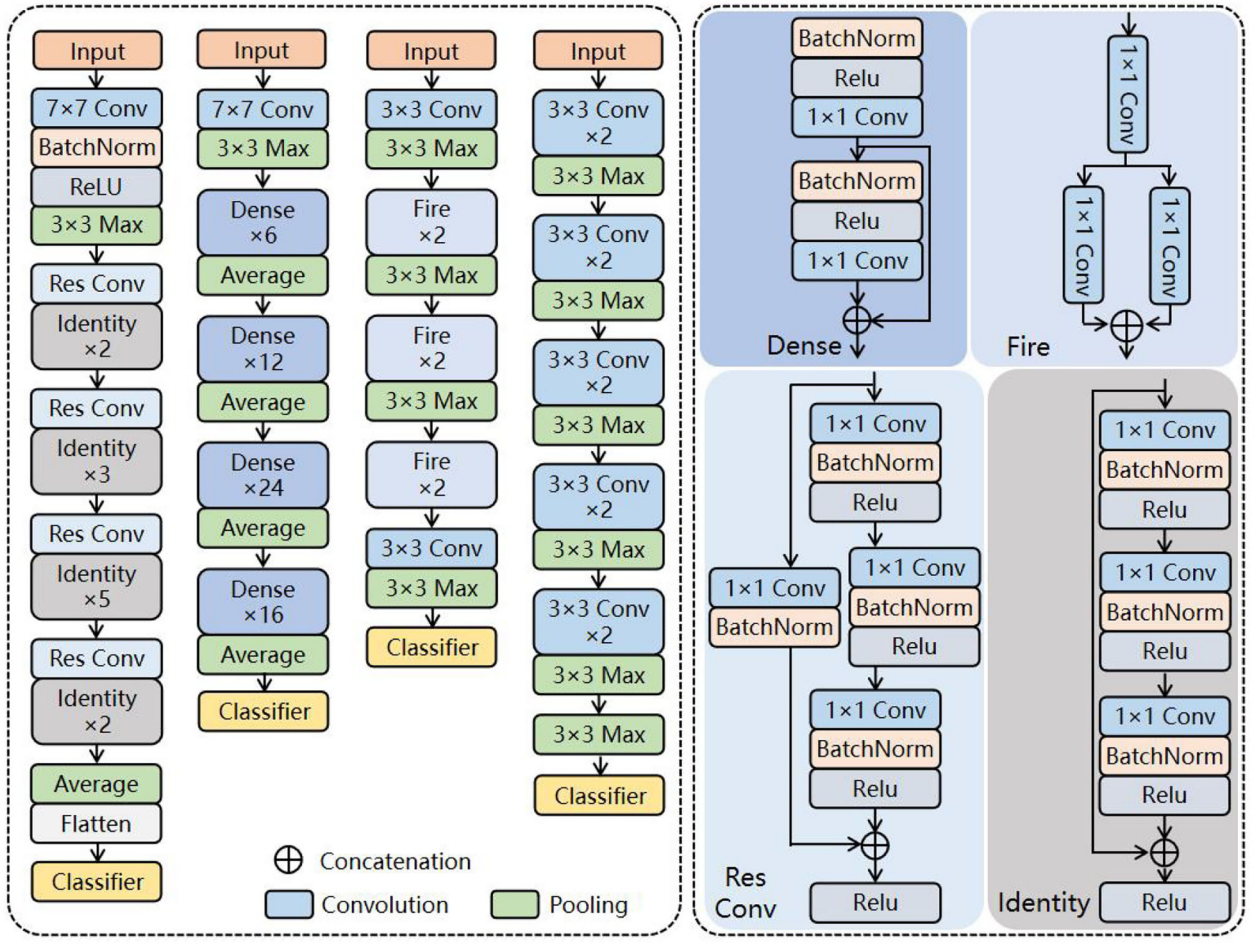
Backbone of proposed architecture.

Here, four CNNs (VGG 19, SqueezeNet, ResNet 50, and DenseNet 121) are adopted to extract the primary features of the fusion image in the proposed architecture. The details are shown in Figure 4.

#### VGG

Visual geometry group has a very systematic architecture. With the deepening of the network, the size of the input image is gradually compressed, but the number of convolution kernels is constantly increasing to explain the reduction in image size. Briefly, abundant 3 × 3 convolutional kernels are accumulated to replace the macrokernels to enhance the depth and width of the network. Thus, the higher the number of activation functions, the richer the extracted features and the stronger the recognition ability of the classification task.

#### SqueezeNet

SqueezeNet replaces the 3 × 3 convolutional kernel with abundant 1 × 1 kernels to reduce the computational cost and accelerate the training process of CNNs, with approximate results of AlexNet on the ImageNet dataset. The network is widely employed for large-scale datasets due to its light weight and high efficiency.

#### ResNet

Different from VGG, ResNet solves the degradation problem of deep networks by connecting the residuals of feature mapping from one layer to the subsequent through residual connections on its basis. Researchers can train deeper networks to improve task representation by solving ill-posed problems.

#### DenseNet

DenseNet, based on ResNet's theory, connects one layer to all subsequent layers by skipping connections, achieving dense skip connections. With further architectural transformations, the internal representation of DenseNet becomes significantly different from ResNets.

One key aspect is the use of network name suffixes in Figure 4. Roughly speaking, the number of layers in the network is represented as “19,” “50,” and “121.” As you can see, the layers of the selected networks range from relatively shallow to extremely deep. This is intentional, as it leads to more architectural diversity.

### Classifier

**Figure 5:**
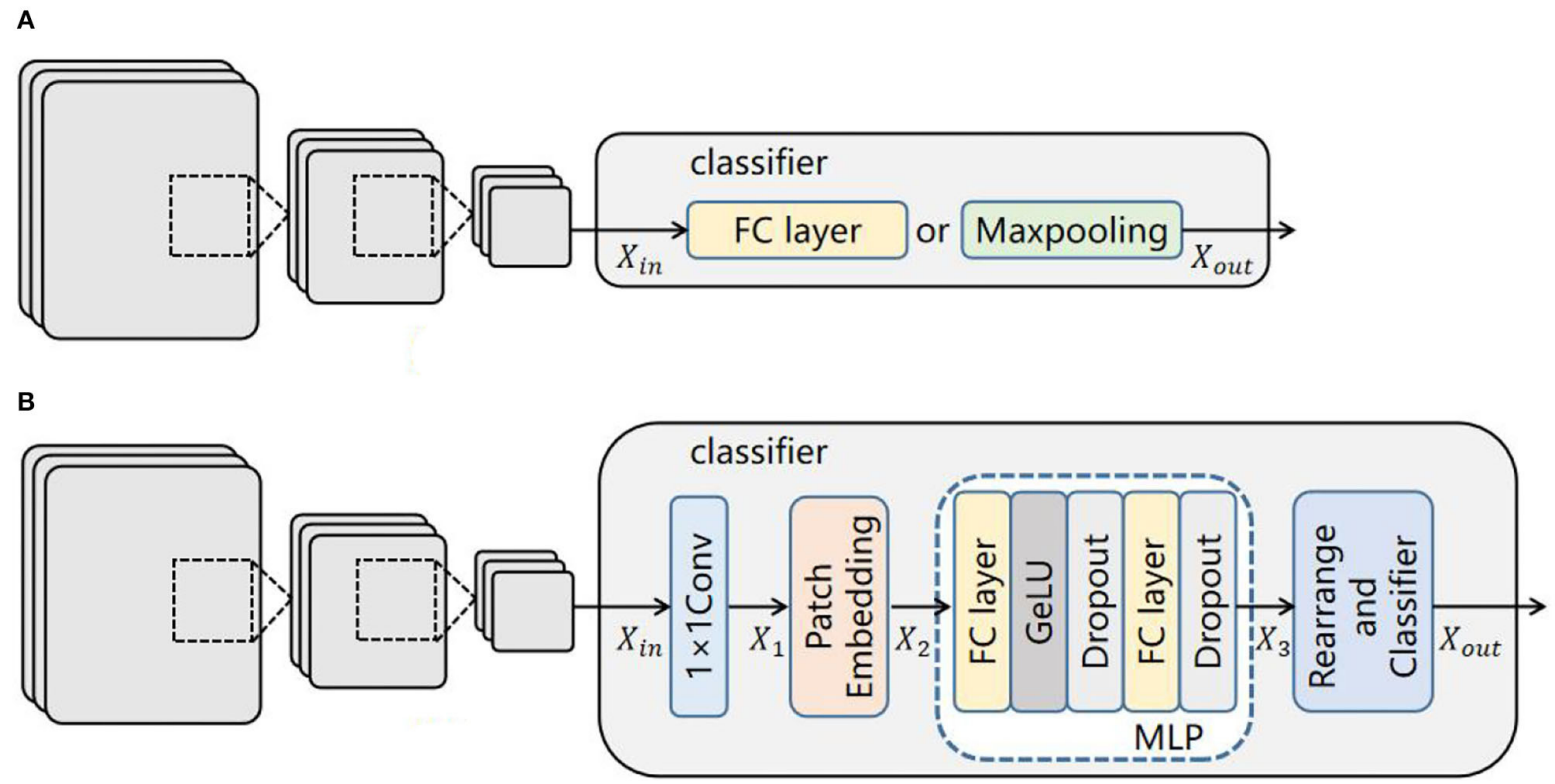
Comparison of classifier structures: **(A)** classifier structure of CNNs; **(B)** our classifier structure.

As the classifiers of CNNs are usually composed of a fully connected layer or maxpooling functions (Figure 5A), they lack the ability to model the long-range dependencies of glaucoma images. Therefore, we propose an effective classifier replacing the originals to offset their limitation in this paper, which is constructed by the MLP of ViT. As mentioned above, ViT can extract the global dependencies, and inspired by (Melas-Kyriazi, 2021), such an ability can be realized by its multilayer perceptron (MLP) alone, so it is employed in our classifier. Figure 5B shows an overview of this module.

First, the input feature map $X_{in}\in {\mathbb{R}}^{H\times W\times C}$ is sent into a 1 × 1 convolutional layer to extract local features and change the dimension to match the next layer. The output of this layer is $X_{1}\in {\mathbb{R}}^{H\times W\times C^{\prime }}$, where (H, W) is the resolution of the initial image, C is the number of initial dimensions, and C ′ is the number of convoluted dimensions.

Second, a patch embedding process including image reshaping and image patch compression is performed. The feature map *X*_1_ is reshaped into an N sequence of flattened 2D patches $X_{p}^{i}\;$(Equation 1):


(1) $$\begin{array}{r} {\text{X}}_{\text{p}}^{\text{i}}=\text{P}\times \text{P}\times \text{C },\text{i}\in \left\{1,2,\cdots \;,\text{N}\right\} \end{array}$$

where (P, P) is the resolution of each image patch, and N = H × W/P2 is the generating number of image patches. Then, $X_{p}^{i}$ is compressed into a D-dimensional embedding space by a trainable linear projection for the MLP layer (Equation 2).


(2) $$\begin{array}{r} {\text{X}}_{2}=\left[{\text{X}}_{\text{P}}^{1}\text{E};{\text{X}}_{\text{P}}^{2}\text{E};\cdots \;;{\text{X}}_{\text{P}}^{\text{N}}\text{E}\right]+{\text{E}}_{\text{pos}}\;\left(2\right) \end{array}$$

where *E* ∈ ℝ^(*P*^2^ × *C*′) × *D*^ is the embedding projection of the patch, $E_{pos}\in {\mathbb{R}}^{N\times D}$ is the positional embedding, and X2 is the encoded image sequence.

Third, the processed data sequence X2 is transferred into the MLP layer (Equations 3, 4).


(3) $$\begin{array}{r} \text{X}2^{\prime }\text{ =Dropout}(\text{Gelu}(\text{FC}(\text{X2})) \end{array}$$

(4) $$\begin{array}{rcl} \text{X}3 & = & \text{Dropout}\left(\text{FC}\left(\text{X}2^{\text{′}}\right)\right) \end{array}$$

where Gelu and Dropout are activation functions used to prevent network overfitting and improve training accuracy. FC is a fully connected layer which transforms the convolution output of the two-dimensional feature map into a one-dimensionalvector.

Finally, the output of the MLP layer is subsequently rearranged to the initial size of the input image $X_{out}\in {\mathbb{R}}^{H\times W\times C}$(Eq. 5), and the glaucoma category is predicted by a classifier.


(5) $$\begin{array}{r} \text{Xout}=\text{rearrange}\left(\text{X}3,\left(\text{hw}\right)\left(\text{p}1\text{p}2\text{c}\right)\to \text{c}\left(\text{hp}1\right)\left(\text{wp}2\right)\right) \end{array}$$

### Evaluation Criteria

To evaluate the effectiveness of the proposed methods, we employ the accuracy, Jaccard score, Kappa score, recall, and F1 score. Accuracy indicates the proportion of the correct sample number in the total sample number. Recall represents the number of samples predicted to be positive out of the total number of true positive samples. The F1 score is the ratio of accuracy to recall. The Jaccard score evaluates the similarity and diversity of samples. The Kappa score assesses the consistency between the predicted classification results and actual results, and we employ it to evaluate the efficiency of multiclassification architectures.


$$\begin{array}{l} \begin{array}{lll} \text{Precision} & = & \frac{\text{TP}}{\text{TP}+\text{FP}}\\ \text{ Recall} & = & \frac{\text{TP}}{\text{TP}+\text{FN}}\\ \text{Jaccard}\;\text{score} & = & \frac{\text{TP}}{\text{TP}+\text{FP}+\text{FN}}\\ \text{ F}1\;\text{Score} & = & \frac{2•\text{precision}•\text{recall}}{\text{precision}+\text{recall}}\\ \text{ Accuracy} & = & \frac{\text{TP}+\text{TN}}{\text{TP}+\text{TN}+\text{FP}+\text{FN}}\\ \;{\text{P}}_{\text{e}} & = & \frac{\left(\text{TP}+\text{FN})(\text{TP}+\text{FP})+(\text{TN}+\text{FN})(\text{TN}+\text{FP}\right)}{{\left(\text{TP}+\text{TN}+\text{FP}+\text{FN}\right)}^{2}}\\ \text{Kappa}\;\text{score} & = & \frac{\text{Accuracy}-{\text{P}}_{\text{e}}}{1-{\text{P}}_{\text{e}}} \end{array} \end{array}$$

where TP is true positive, indicating the number of images correctly classified by the classification algorithm; FN is false negative, indicating the number of images incorrectly classified into other categories by the classification algorithm; TN is true negative, indicating that the classification algorithm correctly classifies non-category images into other categories; and FP is false-positive, indicating that the classification algorithm incorrectly classifies non-category images into such categories.

## Experiment and Discussion

In this section, the experimental setup of our study is introduced. Then, four experiments are conducted to present the effectiveness of our architecture. Finally, the results are shown and discussed in detail.

### Experimental Setup

The experiments are conducted on a server equipped with an NVIDIA GeForce RTX 2060Ti graphic processing unit (GPU) and 16 GB of random-access memory. The compiler is PyCharm, the programming language is Python, and the experimental framework is PyTorch.

In this paper, the adaptive momentum estimation (Adam) optimizer is chosen to update the parameters of the proposed architecture, CrossEntropy Loss is set as the Loss function, and the learning rate is 0.0001. The epochs are set as 60, and the batch size is set as 8. Based on our newly constructed dataset, the proportion of the training set and testing set is set as 8:2; that is, 1,068 fundus and gray scale images are used as the training set, and 268 fundus and gray scale images are used as the testing set.

### Experimental Results and Discussion

#### Comparison of Reconstructed Visual Field Gray Scale Images

In this section, to prove the superiority of the visual field gray scale image being reconstructed at higher resolution proposed in this paper, we conduct experiments on ordinary gray images and newly reconstructed gray scale images based on the proposed architecture. Meanwhile, evaluation criteria are employed to present the whole performance of the proposed multimodal classification architecture. The results are listed in Table 2.

**Table 2:** Comparison of performances before and after reconstructed gray scale image.

	**Ordinary gray image**					**Reconstructed gray scale image**				
	**Accuracy**	**F1 score**	**Kappa**	**Jaccard**	**Recall**	**Accuracy**	**F1 score**	**Kappa**	**Jaccard**	**Recall**
SqueezeNet 1_1	0.772	0.753	0.690	0.623	0.772	0.793	0.779	0.724	0.652	0.793
Vgg 19	0.757	0.749	0.674	0.613	0.757	0.882	0.880	0.842	0.788	0.882
ResNet 50	0.797	0.795	0.729	0.665	0.797	0.918	0.918	0.890	0.849	0.918
DenseNet 121	0.790	0.787	0.720	0.659	0.790	0.888	0.889	0.849	0.803	0.888
Average^*^	0.779	0.771	0.703	0.640	0.779	0.870	0.866	0.826	0.773	0.870

* *Average = average value of above four CNNs*.

Table 2 indicates that the results of using the reconstructed gray scale image are more effective than the common gray scale image. The results of the proposed architecture are enhanced by 9.1, 9.6, 12.3, 13.3, and 9.1% in terms of average accuracy, F1 score, Kappa score, Jaccard score, and recall, respectively, compared with the results of common gray scale images. In particular, the accuracy of this task is enhanced by 12.1% by ResNet50. With these satisfying results, we draw the conclusion that the diagnostic architecture benefits from the reconstruction of the visual field gray scale image at higher resolution.

#### Comparison of Multimodal Fusion

In this section, two experiments are designed to present the effectiveness of multimodal fusion. The fundus image is first individually inputted to the proposed architecture, and then, the fundus image and the reconstructed gray scale image of the visual field are integrated into the multimodal fusion image and sent into the diagnostic architecture. The results are shown in Tables 3, 4. Finally, we compare Tables 2–4 to verify the ability of multimodal fusion in the severity diagnosis of glaucoma.

**Table 3:** Results of fundus images.

	**Accuracy**	**F1 score**	**Kappa**	**Jaccard**	**Recall**
SqueezeNet 1_1	0.696	0.662	0.595	0.528	0.696
Vgg 19	0.704	0.692	0.604	0.559	0.704
ResNet 50	0.687	0.682	0.581	0.534	0.687
DenseNet 121	0.716	0.707	0.622	0.559	0.716
Average	0.701	0.686	0.600	0.545	0.701

**Table 4:** Results of multimodal fusion.

**CNN model**	**Class no**.	**Acc**	**AUC**	**Spec**	**Sen**	**F1**	**Kappa**	**Avg.Acc**	**Avg.F1**	**Avg.AUC**
SqueezeNet1_1	Class 0	0.948	0.965	1.0	0.930	0.909	0.873	0.896	0.895	0.931
	Class 1	0.926	0.866	0.743	0.990	0.839	0.792			
	Class 2	0.948	0.955	0.969	0.942	0.896	0.816			
	Class 3	0.970	0.939	0.879	1.0	0.935	0.916			
VGG 19	Class 0	0.956	0.970	1.0	0.940	0.921	0.890	0.911	0.910	0.956
	Class 1	0.948	0.900	0.800	1.0	0.889	0.856			
	Class 2	0.956	0.971	0.942	1.0	0.914	0.885			
	Class 3	0.963	0.924	0.848	1.0	0.918	0.894			
ResNet 50	Class 0	0.971	0.980	0.900	1.0	0.947	0.927	0.918	0.919	0.953
	Class 1	0.934	0.887	0.923	0.936	0.842	0.801			
	Class 2	0.934	0.963	0.848	0.978	0.897	0.848			
	Class 3	0.971	0.929	1.0	0.964	0.923	0.857			
DenseNet 121	Class 0	0.971	0.980	0.900	1.0	0.947	0.928	0.918	0.920	0.939
	Class 1	0.929	0.871	0.920	0.930	0.821	0.777			
	Class 2	0.907	0.963	0.848	0.936	0.857	0.788			
	Class 3	0.950	0.946	0.862	0.973	0.877	0.846			

By comparing Tables 2–4, the results of multimodal fusion data are better than single-path data: the accuracy of the above four CNNs achieves 89.6, 91.1, 91.8, and 91.8% in Table 5, and the average accuracy with 91.1% is higher than in Table 2 (reconstructed gray scale image) with 87.0% and Table 3 (fundus image) with 70.1%. The proposed architecture is enhanced by 4.5% in terms of the average F1 score compared with the results of the reconstructed gray scale image and 22.5% of the fundus image and improves by 5.4 and 28% in terms of the average kappa score. These results suggest that the proposed multimodal classification architecture is capable of superior diagnosis for glaucoma severity than a single type of data.

**Table 5:** Ablation experiment of data augmentation.

	**Augmentation**	**Accuracy**	**F1 score**	**Kappa**	**Jaccard**	**Recall**
SqueezeNet 1_1	No	0.814	0.811	0.740	0.689	0.814
	Yes	0.896	0.895	0.862	0.812	0.896
Vgg 19	No	0.735	0.720	0.620	0.590	0.735
	Yes	0.911	0.910	0.881	0.836	0.911
ResNet 50	No	0.762	0.762	0.663	0.644	0.762
	Yes	0.918	0.919	0.889	0.852	0.918
DenseNet 121	No	0.812	0.812	0.736	0.699	0.812
	Yes	0.918	0.920	0.889	0.854	0.918

**Figure 6:**
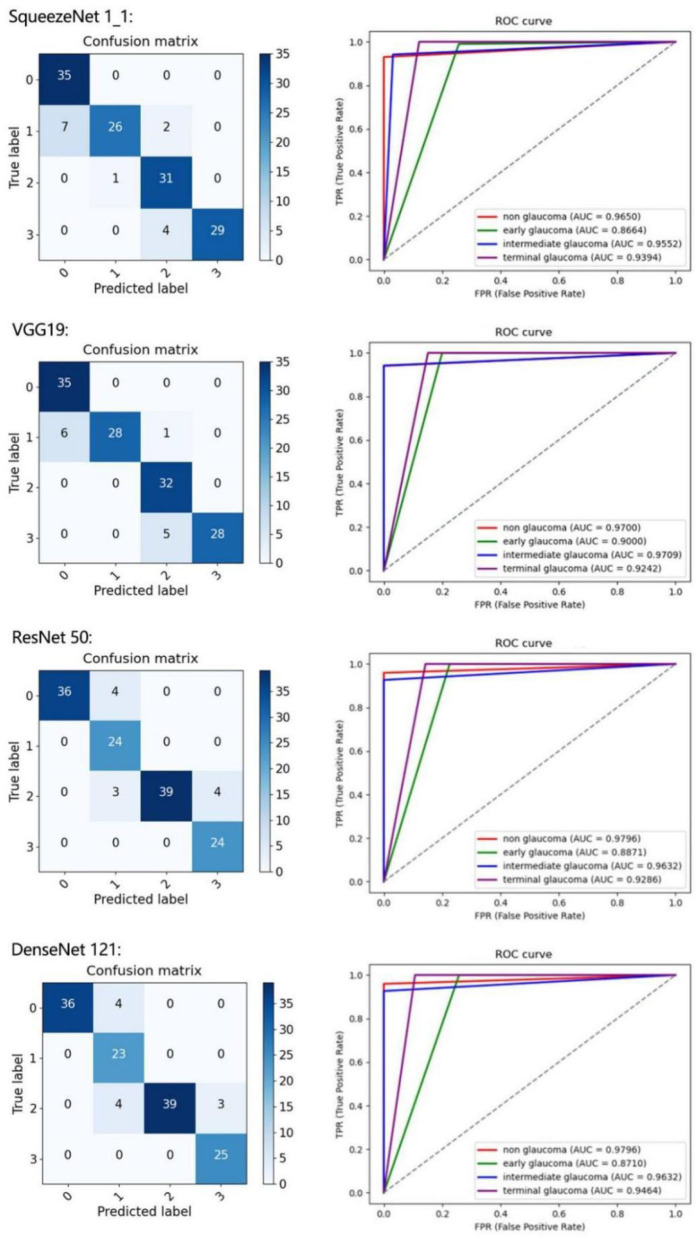
Results of four classes on confusion matrix **(left)** and receiver operating characteristic (ROC) curves **(right)** for SqueezeNet1_1, VGG 19, ResNet 50, and DenseNet 121.

To further present the improvements of the proposed architecture, the classification results of each class are detailed in Table 4. We calculate the confusion matrix, AUC (Figure 6), and values for all the evaluation criteria including accuracy (Acc), sensitivity (Sen), specificity (Spec), Kappa score, and F1-score. Every CNN represents unique performance in the testing of glaucoma data. For instance, using DenseNet 121 led to the highest level of ordered pairs of (i) average accuracy and (ii) average F1-score of 91.8 and 91.2%, respectively, but its average AUC was lower than those of VGG 19 and ResNet 50.

**Figure 7:**
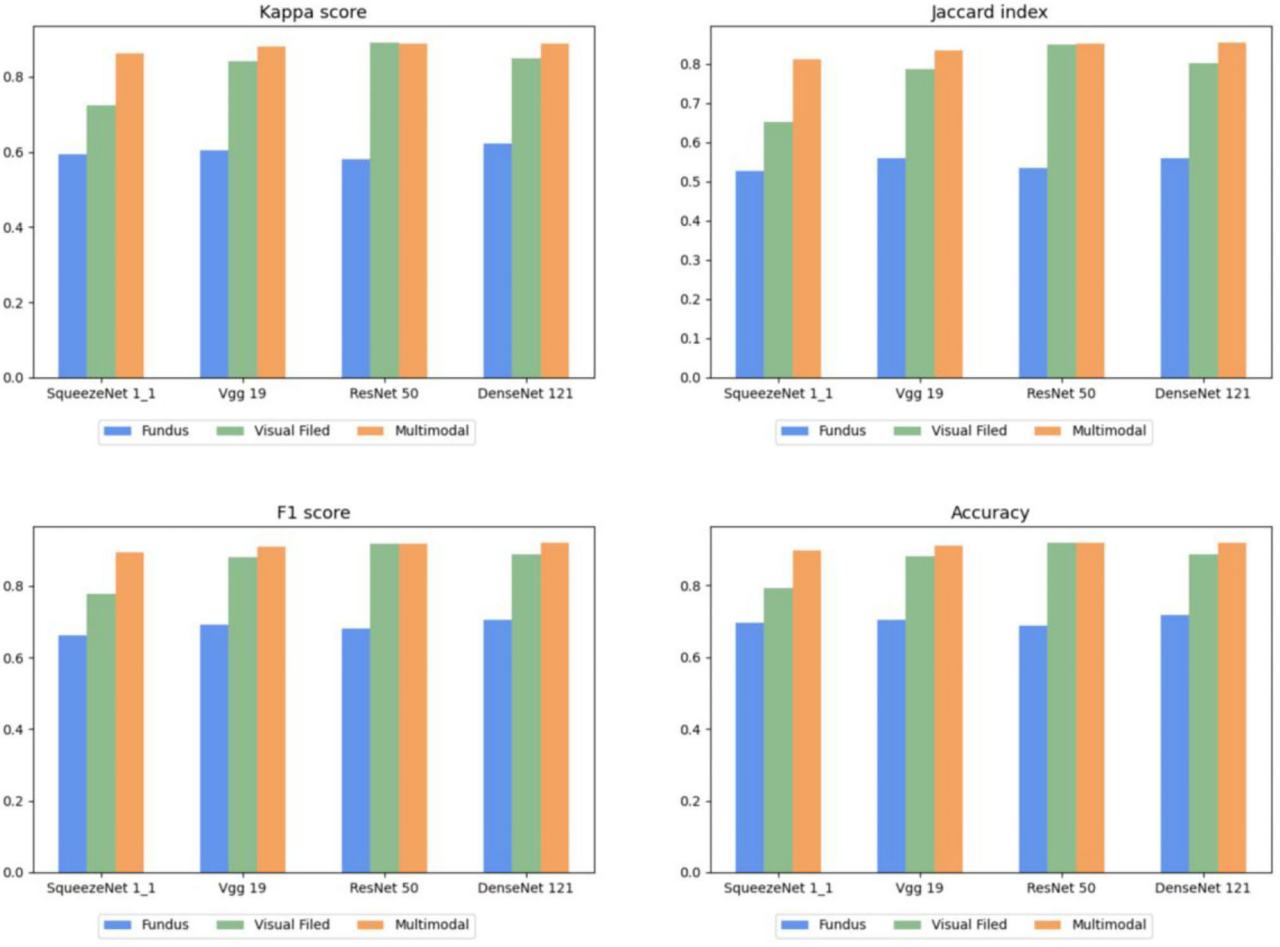
Comparison of multimodal fusion and single path.

To describe this comparison more clearly, the histograms of Tables 2–4 are shown in Figure 7, in which each evaluation metric of different CNNs (SqueezeNet1_1, VGG 19, ResNet 50, and DenseNet 121) is compared. Based on Figure 7, the same conclusion as above can be drawn.

#### Ablation Study

##### Ablation Study of Data Augmentation

In this section, we conduct an ablation experiment to prove the effectiveness of data augmentation technology. The results are shown in Table 5.

Table 5 compares the performance with or without data augmentation, and apparent improvements are obtained in all evaluation criteria. These results demonstrate that data augmentation technology has strong ability in the task of glaucoma classification.

##### Ablation Study of Proposed Classifier

In this section, we conduct an ablation experiment to prove the effectiveness of the proposed classifier, and the results are shown in Table 6.

**Table 6:** Ablation experiment of proposed classifier.

	**Accuracy**	**F1 score**	**Kappa**	**Jaccard**	**Recall**
SqueezeNet 1_1	0.889	0.890	0.853	0.811	0.889
SqueezeNet 1_1+Classifier	0.901	0.900	0.868	0.820	0.901
Vgg 19	0.864	0.863	0.818	0.765	0.864
Vgg 19+Classifier	0.911	0.911	0.881	0.837	0.911
ResNet 50	0.882	0.883	0.851	0.847	0.882
ResNet 50+Classifier	0.924	0.924	0.897	0.862	0.924
DenseNet 121	0.913	0.911	0.886	0.844	0.913
DenseNet 121+Classifier	0.939	0.939	0.917	0.889	0.939

In this section, 5-fold cross-validation is used to evaluate the performance of the proposed classifier in the above CNNs. Table 6 lists the average results of the conducted experiments, which demonstrates that various evaluation metrics of these CNNs are improved to different degrees with the proposed classifier. Furthermore, our classifier can be flexibly plugged into common CNNs to integrate global features of images to enhance the performance in the diagnosis of glaucoma. The same conclusion can be drawn on the combination of multimodal classification architecture and the classifier.

**Figure 8:**
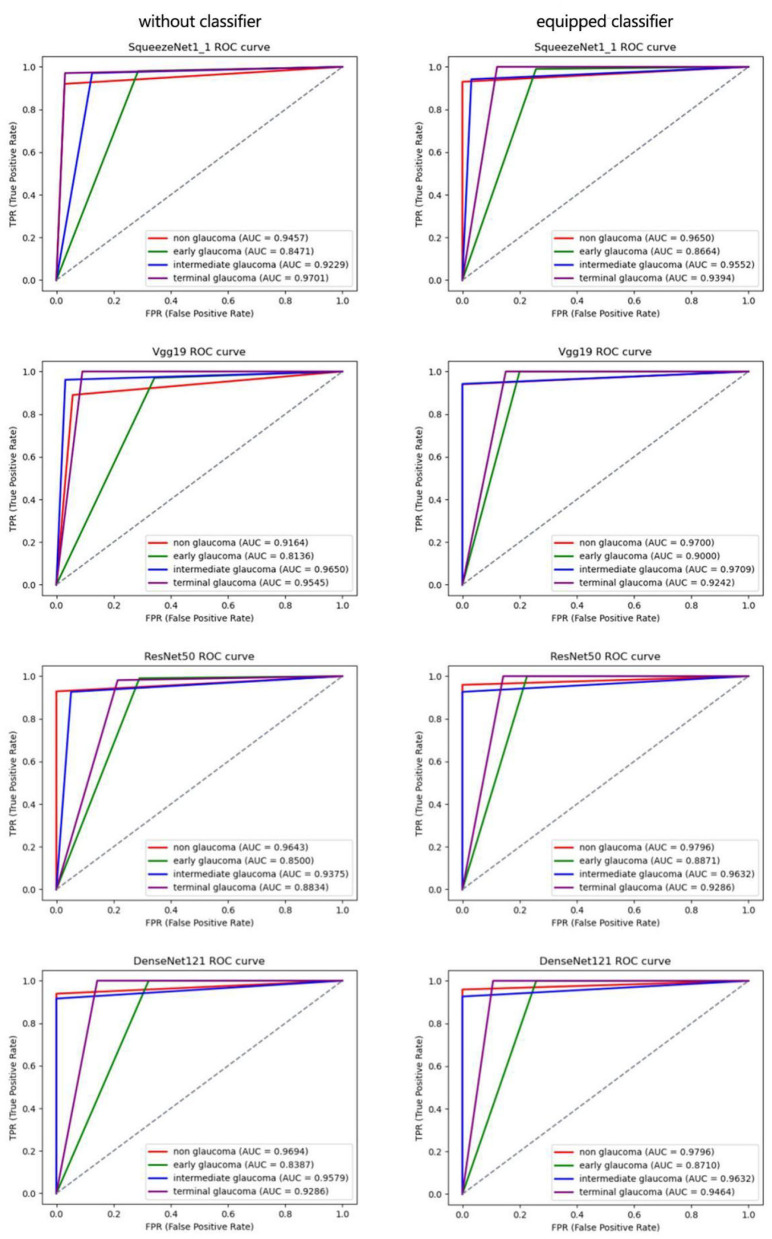
Receiver operating characteristic curves of each subcategory for 4-category classification deep CNN.

To present the efficiency of the proposed classifier more clearly, we use the ROC curve to describe the results of each class in Figure 8. The AUC value can effectively measure the performance of the algorithm, which is defined as the area under the ROC curve. According to Figure 8, the AUC values of normal, early glaucoma, intermediate, and terminal glaucoma are improved to different degrees by each algorithm with the proposed classifier.

#### Comparison of Analogous Approaches

To prove the superiority of the proposed multimodal classification architecture over analogous approaches (Bizios et al., 2011; Chen et al., 2019), we compare the results for the same diagnosis task.

Table 7 shows that the proposed architecture achieves the best results with 0.975 in terms of average accuracy in the classification task of normal and glaucoma. This further demonstrates the advantage of the proposed multimodal classification architecture in glaucoma diagnosis.

**Table 7:** Comparison of analogous approaches.

	**Accuracy**	**AUC**	**Kappa**	**spec**	**Sen**
Bizios et al. (2011)	0.9539	0.978	–	–	–
Chen et al. (2019)	0.9688	0.99	–	1.000	0.9167
Liu et al. (2014)	–	0.869	–	–	–
Ours	0.975	0.992	0.942	0.992	0.957

## Conclusion and Outlook

In this paper, we proposed a multimodal classification architecture based on deep learning for glaucoma severity diagnosis. The advantages of the framework are as follows: (1) More subtle gray scale units and corresponding gray scale images are reconstructed to address the limitation that the inferior resolution of common visual field gray scale images affects feature extraction in the task of glaucoma diagnosis. (2) Fundus images and reconstructed gray scale images of the visual field are fused as multimodal fusion images for the severity classification of glaucoma. Through experiments, we precisely distinguished the severity of glaucoma as normal, early, intermediate, and terminal by the proposed architecture, which yielded a significant contribution in clinical diagnosis. Meanwhile, we can see that the architecture based on the multimodal fusion image performs much better than the single-path architecture, which means that the multimodal fusion input improves the classification ability of the architecture. (3) We proposed a plug-and-play classifier to offset the CNNs' limitation of extracting global sequence information. This significantly improved the architecture's function of feature extraction. Experimental results demonstrated that with our classifier, regardless of what network is chosen as the architecture's backbone, the performance of the architecture is enhanced significantly.

There are many glaucoma patients worldwide, and the detection of the severity is very difficult, which results in a heavy burden and consumes considerable time for ophthalmologists. The proposed diagnosis architecture designed for the severity classification of glaucoma can be very convenient. In the future, we will collect more valid data such as OCT and try to integrate the retinal nerve fiber layer into our architecture to better classify the severity of glaucoma.

## Data Availability Statement

The raw data supporting the conclusions of this article will be made available by the authors, without undue reservation.

## Ethics Statement

This study was reviewed and approved by the Ethics Committee of the First Affiliated Hospital of Kunming Medical University, Kunming, China. Written informed consent was obtained from the individual(s) for the publication of any potentially identifiable images or data included in this article.

## Author Contributions

All authors listed have made a substantial, direct, and intellectual contribution to the work and approved it for publication.

## Funding

This work was supported by the National Natural Science Foundation of China (no. 81960176) and the National Natural Science Foundation of China (no. 82160347).

## Conflict of Interest

The authors declare that the research was conducted in the absence of any commercial or financial relationships that could be construed as a potential conflict of interest.

## Publisher's Note

All claims expressed in this article are solely those of the authors and do not necessarily represent those of their affiliated organizations, or those of the publisher, the editors and the reviewers. Any product that may be evaluated in this article, or claim that may be made by its manufacturer, is not guaranteed or endorsed by the publisher.
